# Supplementary material for: Probiotics for Preventing Necrotizing Enterocolitis in Preterm Infants: A Network Meta-Analysis
Source: Nutrients. 2021 Jan 9;13(1):192. doi: 10.3390/nu13010192 (PMC7827781; doi:10.3390/nu13010192)
Supplement: Supplementary file 1 [file nutrients-13-00192-s001.pdf]

Supplemental Information

1) **Supplemental Section A**
2) **Supplemental Section B**
3) **Supplemental Figure 1**
4) **Supplemental Figure 2**
5) **Supplemental Figure 3**
6) **Supplemental Figure 4**
7) **Supplemental Figure 5**
8) **Supplemental Figure 6**
9) **Supplemental Figure 7**
10) **Supplemental Figure 8**
11) **Supplemental Figure 9**
12) **Supplemental Figure 10**
13) **Supplemental Figure 11**
14) **Supplemental Table 1**
15) **Supplemental Table 2**
16) **Supplemental Table 3**
17) **Supplemental Table 4**

Supplemental Section A. PRISMA NMA Checklist of Items to Include When Reporting A Systematic Review Involving a Network Meta-analysis

Section/Topic	Item #	Checklist Item	Reported on Page #
**TITLE**			
Title	1	Identify the report as a systematic review *incorporating a network meta-analysis (or related form of meta-analysis).*	**p. 1**
**ABSTRACT**			
Structured summary	2	Provide a structured summary including, as applicable:<br>**Background:** main objectives<br>**Methods:** data sources; study eligibility criteria, participants, and interventions; study appraisal; and *synthesis methods, such as network meta-analysis.*<br>**Results:** number of studies and participants identified; summary estimates with corresponding confidence/credible intervals; *treatment rankings may also be discussed. Authors may choose to summarize pairwise comparisons against a chosen treatment included in their analyses for brevity.*<br>**Discussion/Conclusions:** limitations; conclusions and implications of findings.<br>**Other:** primary source of funding; systematic review registration number with registry name.	**p. 1**
**INTRODUCTION**			
Rationale	3	Describe the rationale for the review in the context of what is already known, *including mention of why a network meta-analysis has been conducted.*	**p. 1**
Objectives	4	Provide an explicit statement of questions being addressed, with reference to participants, interventions, comparisons, outcomes, and study design (PICOS).	**p. 2**
**METHODS**			
Protocol and registration	5	Indicate whether a review protocol exists and if and where it can be accessed (e.g., Web address); and, if available, provide registration information, including registration number.	**NA**
Eligibility criteria	6	Specify study characteristics (e.g., PICOS, length of follow-up) and report characteristics (e.g., years considered, language, publication status) used as criteria for eligibility, giving rationale. *Clearly describe eligible treatments included in the treatment network, and note whether any have been clustered or merged into the same node (with justification).*	**p. 2**
Information sources	7	Describe all information sources (e.g., databases with dates of coverage, contact with study authors to identify additional studies) in the search and date last searched.	**p. 2**
Search	8	Present full electronic search strategy for at least one database, including any limits used, such that it could be repeated.	**Appendix B**
Study selection	9	State the process for selecting studies (i.e., screening, eligibility, included in systematic review, and, if applicable, included in the meta-analysis).	**Figure 1**
Data collection process	10	Describe method of data extraction from reports (e.g., piloted forms, independently, in duplicate) and any processes for	**pp. 2-3**

		obtaining and confirming data from investigators.	
Data items	11	List and define all variables for which data were sought (e.g., PICOS, funding sources) and any assumptions and simplifications made.	**pp. 2-3**
**Geometry of the network**	**S1**	Describe methods used to explore the geometry of the treatment network under study and potential biases related to it. This should include how the evidence base has been graphically summarized for presentation, and what characteristics were compiled and used to describe the evidence base to readers.	**p. 3**
Risk of bias within individual studies	12	Describe methods used for assessing risk of bias of individual studies (including specification of whether this was done at the study or outcome level), and how this information is to be used in any data synthesis.	**p. 3**
Summary measures	13	State the principal summary measures (e.g., risk ratio, difference in means). *Also describe the use of additional summary measures assessed, such as treatment rankings and surface under the cumulative ranking curve (SUCRA) values, as well as modified approaches used to present summary findings from meta-analyses.*	**p. 3**
Planned methods of analysis	14	Describe the methods of handling data and combining results of studies for each network meta-analysis. This should include, but not be limited to:<br>• *Handling of multi-arm trials;*<br>• *Selection of variance structure;*<br>• *Selection of prior distributions in Bayesian analyses; and*<br>• *Assessment of model fit.*	**p.3<br>Appendix C**
**Assessment of Inconsistency**	**S2**	Describe the statistical methods used to evaluate the agreement of direct and indirect evidence in the treatment network(s) studied. Describe efforts taken to address its presence when found.	**p.3<br>Appendix C**
Risk of bias across studies	15	Specify any assessment of risk of bias that may affect the cumulative evidence (e.g., publication bias, selective reporting within studies).	**p. 3**
Additional analyses	16	Describe methods of additional analyses if done, indicating which were pre-specified. This may include, but not be limited to, the following:<br>• Sensitivity or subgroup analyses;<br>• Meta-regression analyses;<br>• *Alternative formulations of the treatment network; and*<br>• *Use of alternative prior distributions for Bayesian analyses (if applicable).*	**pp. 2-3, Appendix C**
**RESULTS**			
Study selection	17	Give numbers of studies screened, assessed for eligibility, and included in the review, with reasons for exclusions at each stage, ideally with a flow diagram.	**pp. 3-4**
**Presentation of network structure**	**S3**	Provide a network graph of the included studies to enable visualization of the geometry of the treatment network.	**pp. 3-4, Figure 1**

**Summary of network geometry**	**S4**	Provide a brief overview of characteristics of the treatment network. This may include commentary on the abundance of trials and randomized patients for the different interventions and pairwise comparisons in the network, gaps of evidence in the treatment network, and potential biases reflected by the network structure.	**pp. 3-4**
Study characteristics	18	For each study, present characteristics for which data were extracted (e.g., study size, PICOS, follow-up period) and provide the citations.	Table 1
Risk of bias within studies	19	Present data on risk of bias of each study and, if available, any outcome level assessment.	**p. 5**
Results of individual studies	20	For all outcomes considered (benefits or harms), present, for each study: 1) simple summary data for each intervention group, and 2) effect estimates and confidence intervals. *Modified approaches may be needed to deal with information from larger networks.*	***Table 1***
Synthesis of results	21	Present results of each meta-analysis done, including confidence/credible intervals. *In larger networks, authors may focus on comparisons versus a particular comparator (e.g. placebo or standard care), with full findings presented in an appendix. League tables and forest plots may be considered to summarize pairwise comparisons.* If additional summary measures were explored (such as treatment rankings), these should also be presented.	**pp. 3-8, Figure 2-5 Supplemental Figures**
**Exploration for inconsistency**	**S5**	Describe results from investigations of inconsistency. This may include such information as measures of model fit to compare consistency and inconsistency models, *P* values from statistical tests, or summary of inconsistency estimates from different parts of the treatment network.	**Supplemental Figure5**
Risk of bias across studies	22	Present results of any assessment of risk of bias across studies for the evidence base being studied.	**Supplemental Figure1**
Results of additional analyses	23	Give results of additional analyses, if done (e.g., sensitivity or subgroup analyses, meta-regression analyses, *alternative network geometries studied, alternative choice of prior distributions for Bayesian analyses*, and so forth).	**pp. 3-8**
**DISCUSSION**			
Summary of evidence	24	Summarize the main findings, including the strength of evidence for each main outcome; consider their relevance to key groups (e.g., healthcare providers, users, and policy-makers).	**pp. 8-9**
Limitations	25	Discuss limitations at study and outcome level (e.g., risk of bias), and at review level (e.g., incomplete retrieval of identified research, reporting bias). *Comment on the validity of the assumptions, such as transitivity and consistency. Comment on any concerns regarding network geometry (e.g., avoidance of certain comparisons).*	**pp. 8-9**
Conclusions	26	Provide a general interpretation of the results in the context of other evidence, and implications for future research.	**p. 9**
**FUNDING**			

Funding	27	Describe sources of funding for the systematic review and other support (e.g., supply of data); role of funders for the systematic review. This should also include information regarding whether funding has been received from manufacturers of treatments in the network and/or whether some of the authors are content experts with professional conflicts of interest that could affect use of treatments in the network.	**p. 9**

Supplemental Section B Search strategy

Search strings

Pubmed search string
((preterm infant OR pre-term infant) OR (preterm infants OR pre-term infants) OR (preterm neonate OR pre-term neonate) OR (preterm neonates OR pre-term neonates) OR (preterm newborn OR pre-term newborn) OR (preterm newborns OR pre-term newborns) OR (premature infant OR premature infants) OR (premature neonate OR premature neonates) OR (premature newborn OR premature newborns) OR infant, extremely premature [MH] OR premature birth [MH] OR infant, low birth weight [MH] OR infant, very low birth weight [MH]) AND ((necrotizing enterocolitis OR necrotizing entero-colitis) OR (necrot* AND (enterocoli* OR entero-coli*) OR ("necrotizing" OR "entero-colitis" OR "enterocolitis") AND NEC) AND (probiotic OR probiotics OR pro-biotic OR pro-biotics OR probio*) NOT (animals [MH] NOT humans [MH])

Cochrane Library search criteria
preterm infant OR pre-term infant OR preterm infants OR pre-term infants OR preterm neonate OR pre-term neonate OR preterm neonates OR pre-term neonates OR preterm newborn OR pre-term newborn OR preterm newborns OR pre-term newborns OR premature infant OR premature infants OR premature neonate OR premature neonates OR premature newborn OR premature newborns
necrotizing enterocolitis OR necrotizing entero-colitis OR NEC
probiotic OR probiotics OR pro-biotic OR pro-biotics OR probio*

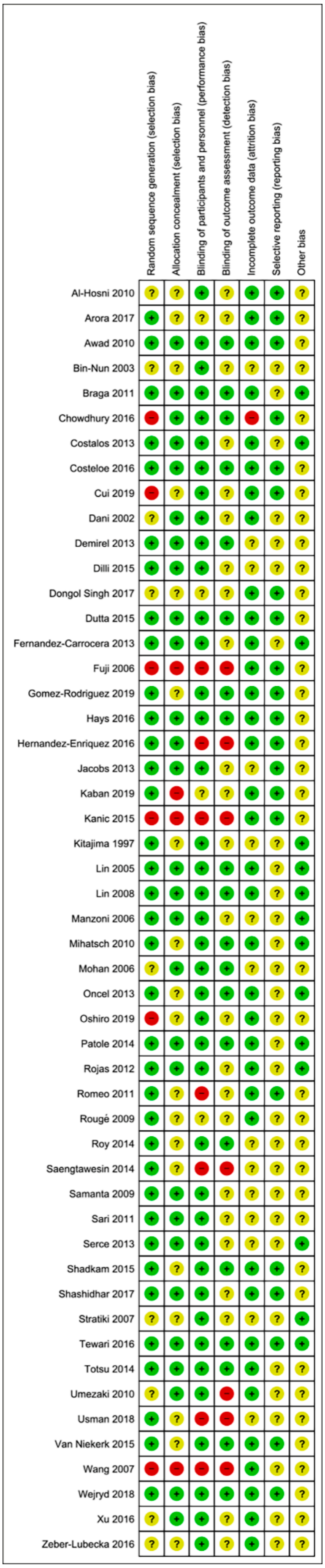

Supplemental Figure 1. Risk of Bias summary.

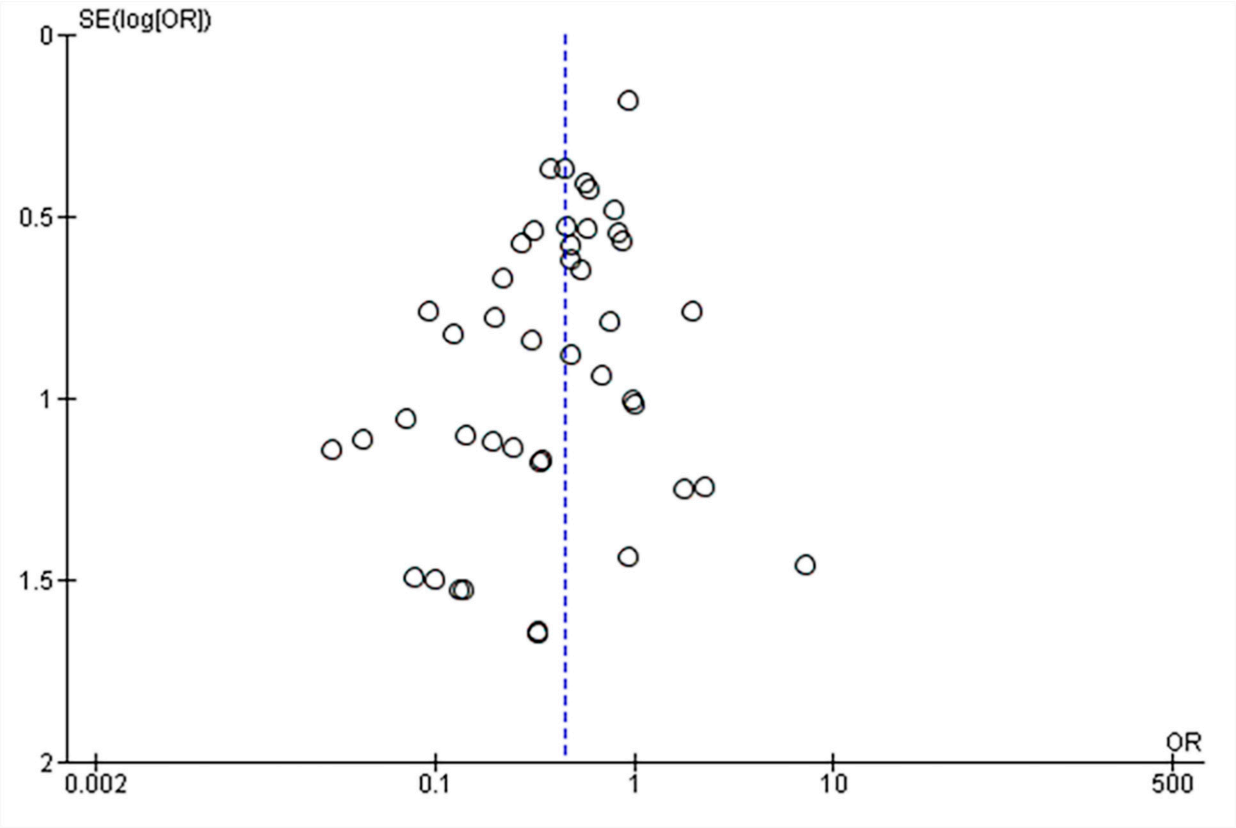

Supplemental Figure 2. Funnel plot showing no clear visual asymmetry.

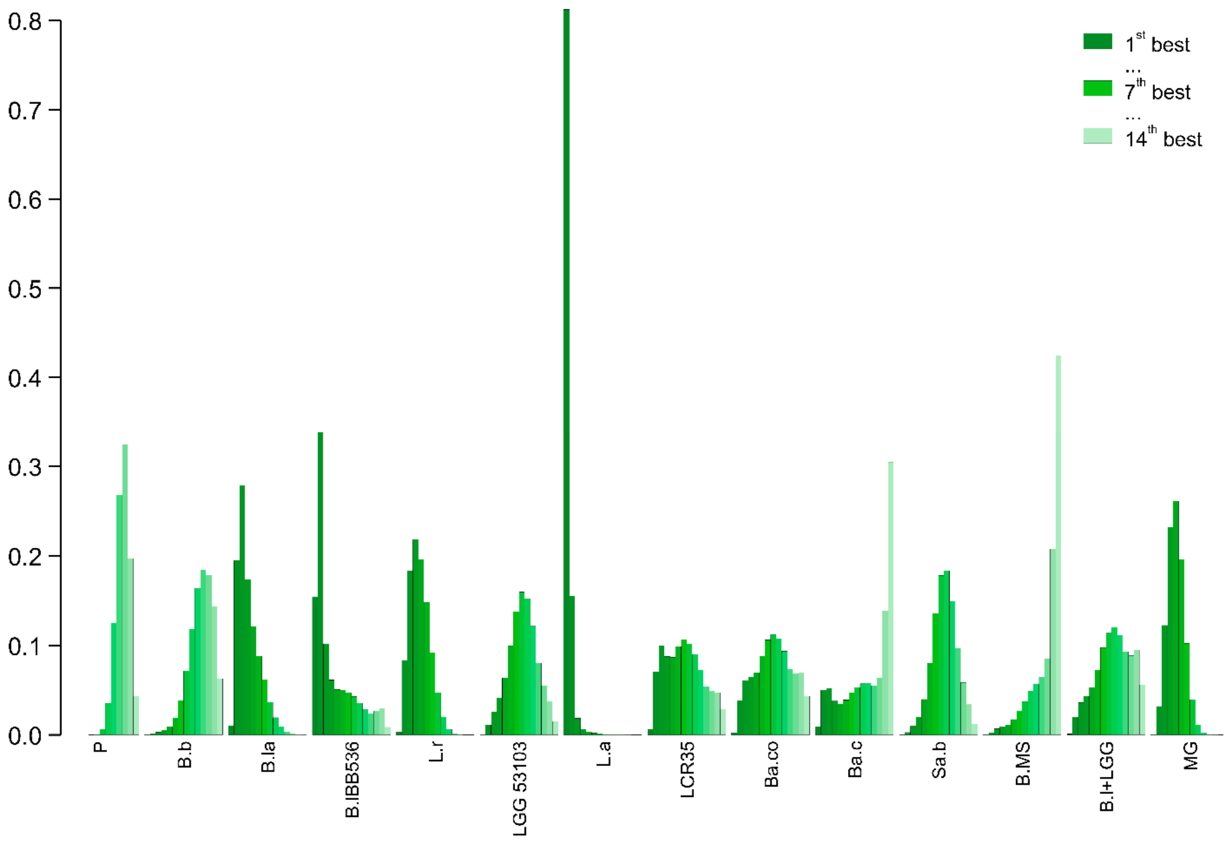

Supplemental Figure 3. Probability bars (rankograms) reporting the probability that each treatment is ranked first, second and so on until fourteenth, for efficacy in the prevention of necrotizing enterocolitis in preterm infants. P: Placebo; B.b: *B. breve* BBG YIT4010, *B. breve* BBG-001, *B. breve* M-16V; B.la: *Bifidobacterium lactis* Bb-12 OR B94; B.lBB536: *B. longum* BB536; L.r: *Lactobacillus reuteri* DSM 17938, *L. reuteri* ATCC 55730; LGG53103: *L. rhamnosus* GG ATCC 53103; L.a: *L. acidophilus* LB; LCR35: *L. casei* var. *rhamnosus* (LCR 35); Ba.co: *Bacillus coagulans* (*L. sporogenes*); Ba.c: *Ba. clausii* (four strains); Sa.b: *Saccharomyces boulardii* CNCM I-745, *Sa. boulardii* CNCMI-3799; B.MS: *B. lactis* Bb-12 + *B. longum* BB536; B.l + LGG: *B. longum* 35,624 + *L. rhamnosus* GG, *B. longum* BB536 + *L. rhamnosus* GG; MG: multi-genus probiotic group.

Exp. \ Ref.	P	B.b	B.la	B.lBB536	L.r	LGG 53103	L.a	LCR35	Ba.co	Ba.c	Sa.b	B.MS	B.l+LGG	MG
P	1.00													
B.b	0.89 (0.38-1.86)	1.00												
B.la	0.26 (0.10-0.63)*	0.29 (0.09-1.02)	1.00											
B.lBB536	0.20 (0.01-1.64)	0.22 (0.01-2.16)	0.74 (0.02-6.90)	1.00										
L.r	0.32 (0.16-0.57)*	0.36 (0.13-0.93)*	1.20 (0.38-3.59)	1.62 (0.17-52.2)	1.00									
LGG 53103	0.58 (0.23-1.37)	0.66 (0.20-2.13)	2.22 (0.62-7.70)	3.01 (0.29-102)	1.84 (0.63-5.63)	1.00								
L.a	0.03 (0.00-0.21)*	0.04 (0.00-0.29)*	0.12 (0.01-1.01)	0.17 (0.01-8.30)	0.10 (0.01-0.78)*	0.06 (0.00-0.46)*	1.00							
LCR35	0.47 (0.11-1.80)	0.53 (0.11-2.59)	1.79 (0.33-8.97)	2.46 (0.19-94.9)	1.48 (0.33-6.98)	0.80 (0.15-4.11)	14.54 (1.35-260)†	1.00						
Ba.co	0.57 (0.15-2.02)	0.64 (0.14-2.94)	2.17 (0.43-10.4)	2.98 (0.23-117)	1.79 (0.43-7.91)	0.98 (0.20-4.71)	17.56 (1.74-306)†	1.22 (0.18-8.06)	1.00					
Ba.c	1.00 (0.09-11.1)	1.13 (0.09-14.4)	3.82 (0.30-49.8)	5.53 (0.21-317)	3.17 (0.27-40.1)	1.72 (0.14-22.5)	31.97 (1.47-1022)†	2.14 (0.14-34.7)	1.76 (0.12-27.9)	1.00				
Sa.b	0.62 (0.30-1.30)	0.70 (0.25-2.16)	2.38 (0.76-7.64)	3.24 (0.33-107)	1.97 (0.79-5.58)	1.08 (0.35-3.46)	19.18 (2.54-283)†	1.34 (0.29-6.51)	1.10 (0.25-5.06)	0.63 (0.05-7.65)	1.00			
B.MS	1.46 (0.31-6.73)	1.65 (0.30-9.36)	5.55 (1.07-29.4)†	7.49 (0.86-242)	4.64 (0.91-25.4)	2.53 (0.44-14.9)	45.53 (4.03-875)†	3.14 (0.41-24.5)	2.59 (0.35-19.5)	1.47 (0.08-24.8)	2.34 (0.42-12.8)	1.00		
B.l+LGG	0.66 (0.19-2.10)	0.74 (0.18-3.11)	2.52 (0.56-11.1)	3.47 (0.29-125)	2.10 (0.55-8.23)	1.14 (0.26-4.96)	20.42 (2.14-340)†	1.42 (0.23-8.68)	1.17 (0.20-6.85)	0.65 (0.04-9.44)	1.06 (0.25-4.18)	0.45 (0.06-3.06)	1.00	
MG	0.34 (0.22-0.48)*	0.38 (0.16-0.92)*	1.27 (0.48-3.44)	1.71 (0.20-55.0)	1.06 (0.52-2.31)	0.58 (0.22-1.53)	10.17 (1.53-141)†	0.72 (0.17-3.04)	0.59 (0.15-2.34)	0.34 (0.03-3.76)	0.54 (0.23-1.20)	0.23 (0.05-1.10)	0.51 (0.15-1.80)	1.00

Supplemental Figure 4. Matrix of all treatment comparison estimates, presented as posterior medians of odds ratios from the network meta-analysis with 95% credible intervals. The upper triangle is not displayed to avoid redundancy.

* 97.5% of the posterior distribution is below one (lower risk than the reference).
† 97.5% of the posterior distribution is above one (higher risk than the reference).

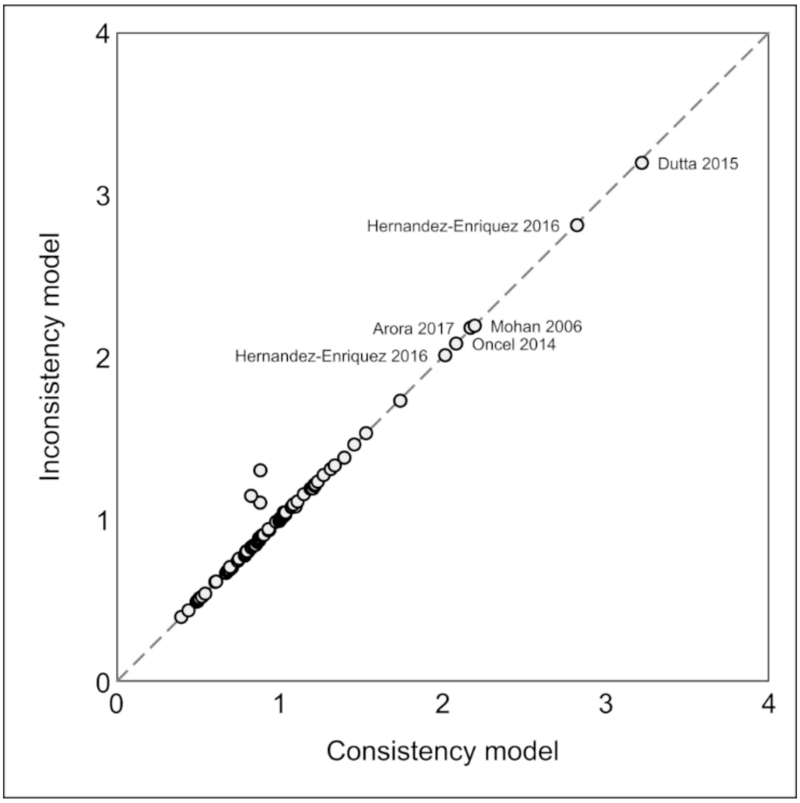

Supplemental Figure 5. Plot of the individual data points' posterior mean deviance contributions for the consistency model (horizontal axis) and the inconsistency model (vertical axis) along with the line of equality. Points that have a low fit are marked with the trial label. The strong similarity between trial-arms deviance contributions as well as between the deviance information criterions of the two models (consistency DIC = 425.10; inconsistency DIC = 426.57), suggests no evidence of inconsistency in the network.

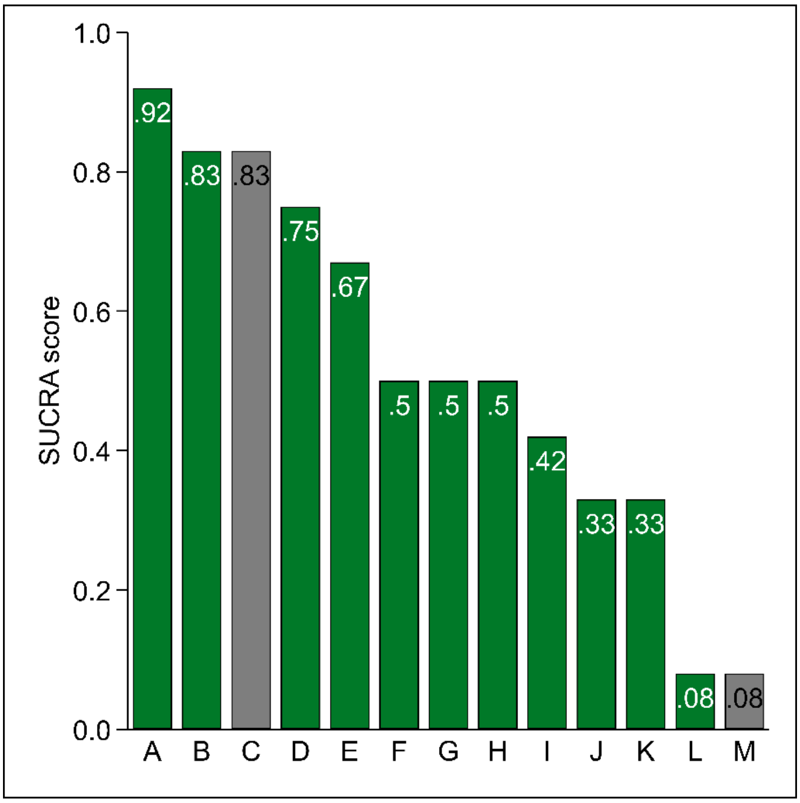

Supplemental Figure 6. Bar chart of SUCRA scores (surface under the cumulative ranking) resulting from a subgroup network meta-analysis conducted on trials that assessed multi-genus (MG) treatments. Non-MG probiotics (treatment comparators) are marked in gray. *B.infantis* PTA-5843 + *E.faecium* PTA-5844 + *L.gasseri* PTA-5845, *B.breve* + *L.casei, L.rhamnosus* GG + *L.paracasei* + *L.casei* + *L.acidophilus* + *Lactococcus lactis* + *B.bifidum* + *B.longum* + *B.infantis* and *B.infantis* ATCC15697 + *L.acidophilus* ATCC4356 reported the best SUCRA values among the evaluated treatments included in the multi-genus probiotic group.

A, *B. infantis* PTA-5843 + *E. faecium* PTA-5844 + *L. gasseri* PTA-5845;
B, *B. breve* + *L. casei;*
C, *L. acidophilus* LB;
D, *L. rhamnosus* GG + *L. paracasei* + *L. casei* + *L. acidophilus* + *Lactococcus lactis* + *B. bifidum* + *B. longum* + *B. infantis;*
E, *B. infantis* ATCC 15697 + *L. acidophilus* ATCC 4356;
F, *B. bifidum* + *B. infantis* + *B. longum* + *L. acidophilus;*
G, *B. bifidum* NCDO 1453 + *L. acidophilus* NCDO 1748;
H, *B. infantis* Bb-02 + *B. lactis* Bb-12 +*S. thermophilus* TH-4;
I, *B. lactis* Bb-12 + *L. rhamnosus* GG;
J, *B. longum* R00175 + *L. helveticus* R0052 + *L. rhamnosus* R0011 + *Sa. boulardii* CNCM I-1079;
K, *L. acidophilus* + *L. rhamnosus* + *L. casei* + *L. plantarum* + *B. infantis* + *S. thermophilus;*
L, *B. bifidum* + *B. lactis* + *B. longum* + *L. acidophilus;*
M, Placebo.

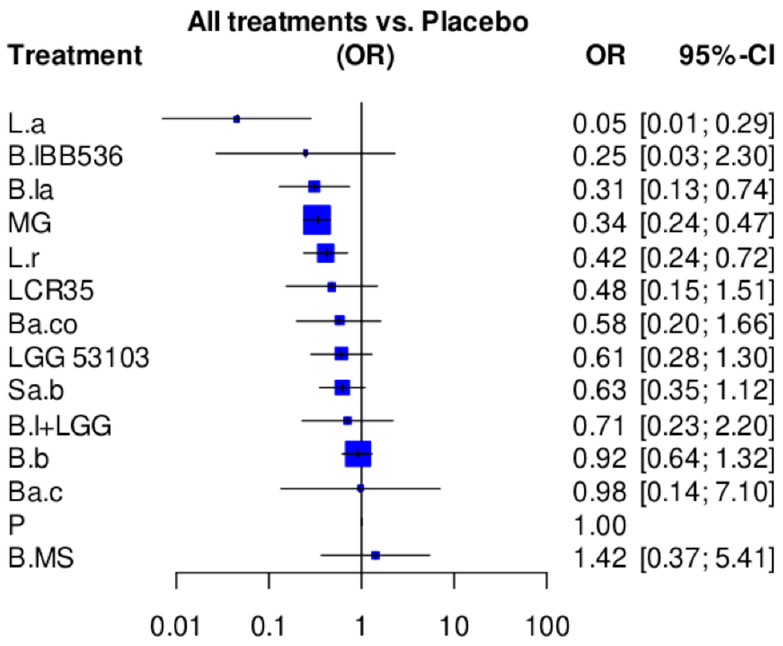

Supplemental Figure 7. Results from frequentist network meta-analysis based on electrical network theory. Forest plot of relative effect sizes compared to placebo for each treatment under study.

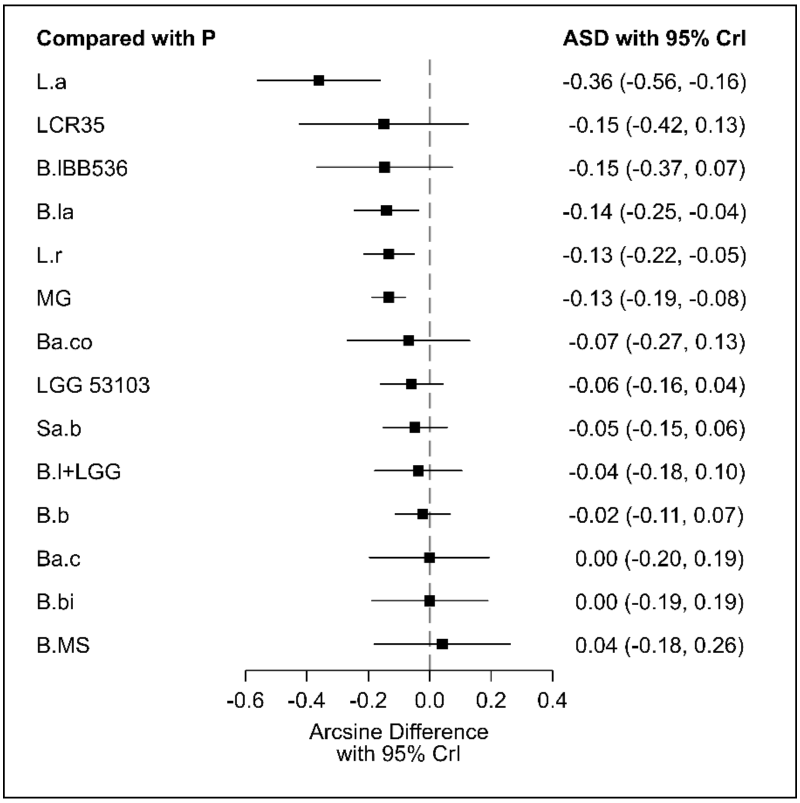

Supplemental Figure 8. Forest plot of relative effect sizes expressed as the arcsine difference (ASD) in the risk of NEC between each treatment and placebo. Nine double-zero studies are now included in the network meta-analysis (Fujii 2006, Kitajima 1997, Oshiro 2019, Romeo 2011, Totsu 2014, Umezaki 2010, Wang 2007, Xu 2016, Zeber-Lubecka 2016), as well as an additional treatment investigated by Totsu and colleagues (*B. bifidum* OLB6378, corresponding to B.bi category).

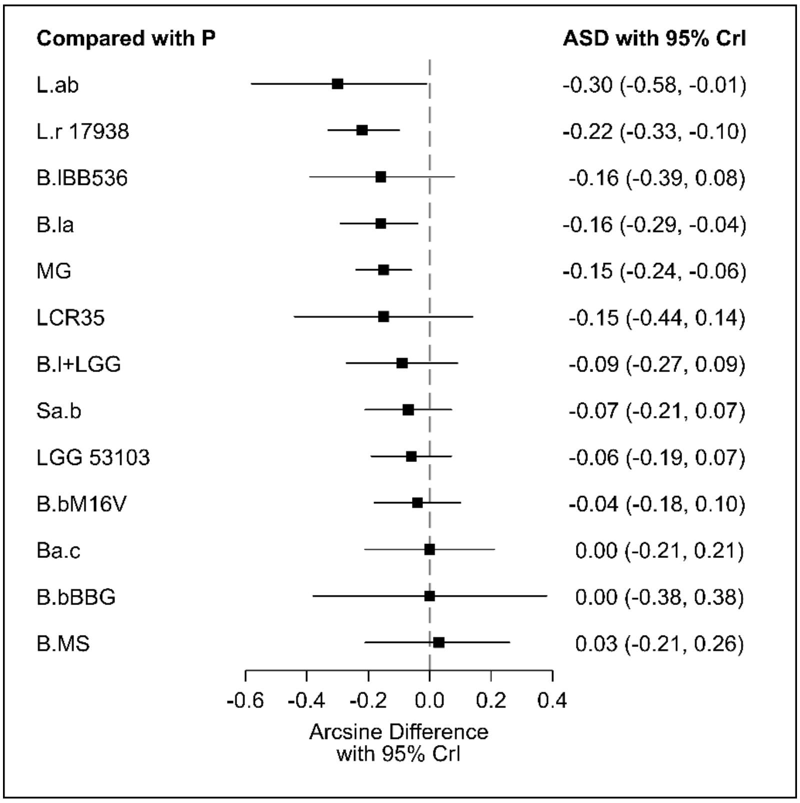

Supplemental Figure 9. Forest plot of relative effect sizes expressed as the arcsine difference (ASD) between the risk of NEC between each treatment and placebo. Only trials that made information on type of feeding available are investigated. Four double-zero studies are now included in the Bayesian meta-analysis (Oshiro 2019, Umezaki 2010, Wang 2007, Xu 2016), as well as an additional treatment investigated by Oshiro and colleagues (B.bBBG, *B. breve* BBG-001).

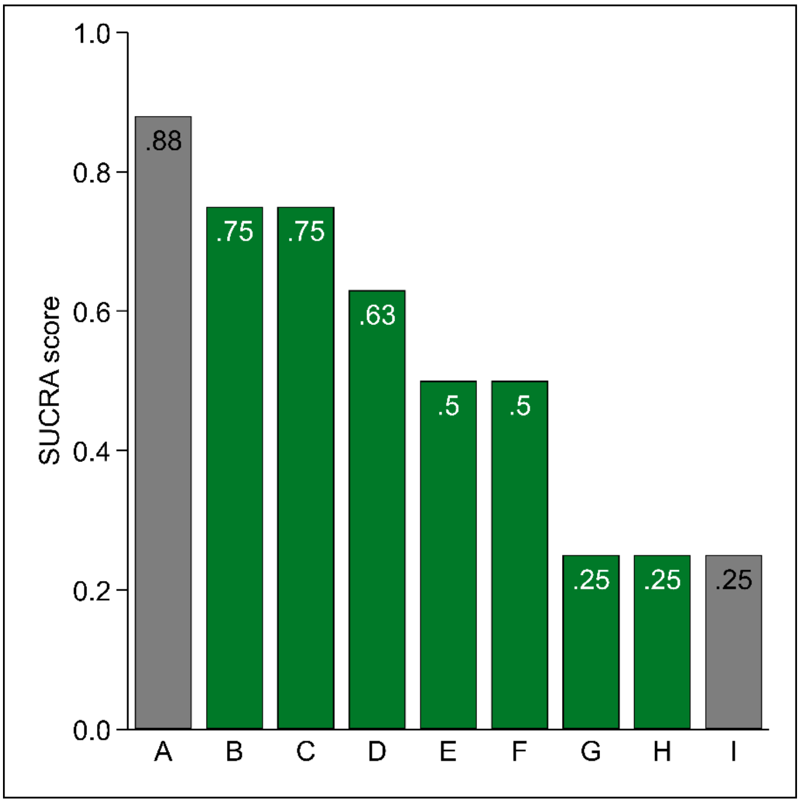

Supplemental Figure 10. Bar chart of SUCRA scores (surface under the cumulative ranking) resulting from secondary network meta-analysis conducted on trials that assessed multi-genera (MG) treatments and provided outcome according to infant feeding. Non-MG probiotics (treatment comparators) are marked in gray. *L.rhamnosus* GG + *L.paracasei* + *L.casei* + *L.acidophilus* + *Lactococcus lactis* + *B.bifidum* + *B.longum* + *B.infantis*, *B.longum* R00175 + *L.helveticus* R0052 + *L.rhamnosus* R0011 + *Sa.boulardii* CNCM-I-1079 and *B.infantis* ATCC15697 + *L.acidophilus* ATCC4356 reported the best SUCRA values among the evaluated treatments in the multi-genus probiotic group.

A, *Lactobacillus acidophilus* LB;

B, *L. rhamnosus* GG + *L. paracasei* + *L. casei* + *L. acidophilus* + *Lactococcus lactis* + *B. bifidum* + *B. longum* + *B. infantis*;

C, *B. longum* R00175 + *L. helveticus* R0052 + *L. rhamnosus* R0011 + *Sa. boulardii* CNCM I-1079;

D, *B. infantis* ATCC 15697 + *L. acidophilus* ATCC 4356;

E, *B. bifidum* + *B. infantis* + *B. longum* + *L. acidophilus*;

F, *La. acidophilus* + *L. rhamnosus* + *L. casei* + *L. plantarum* + *B. infantis* + *S. thermophilus*;

G, *B. bifidum* NCDO 1453 + *L. acidophilus* NCDO 1748;

H, *B. bifidum* + *B. lactis* + *B. longum* + *L. acidophilus*;

I, Placebo.

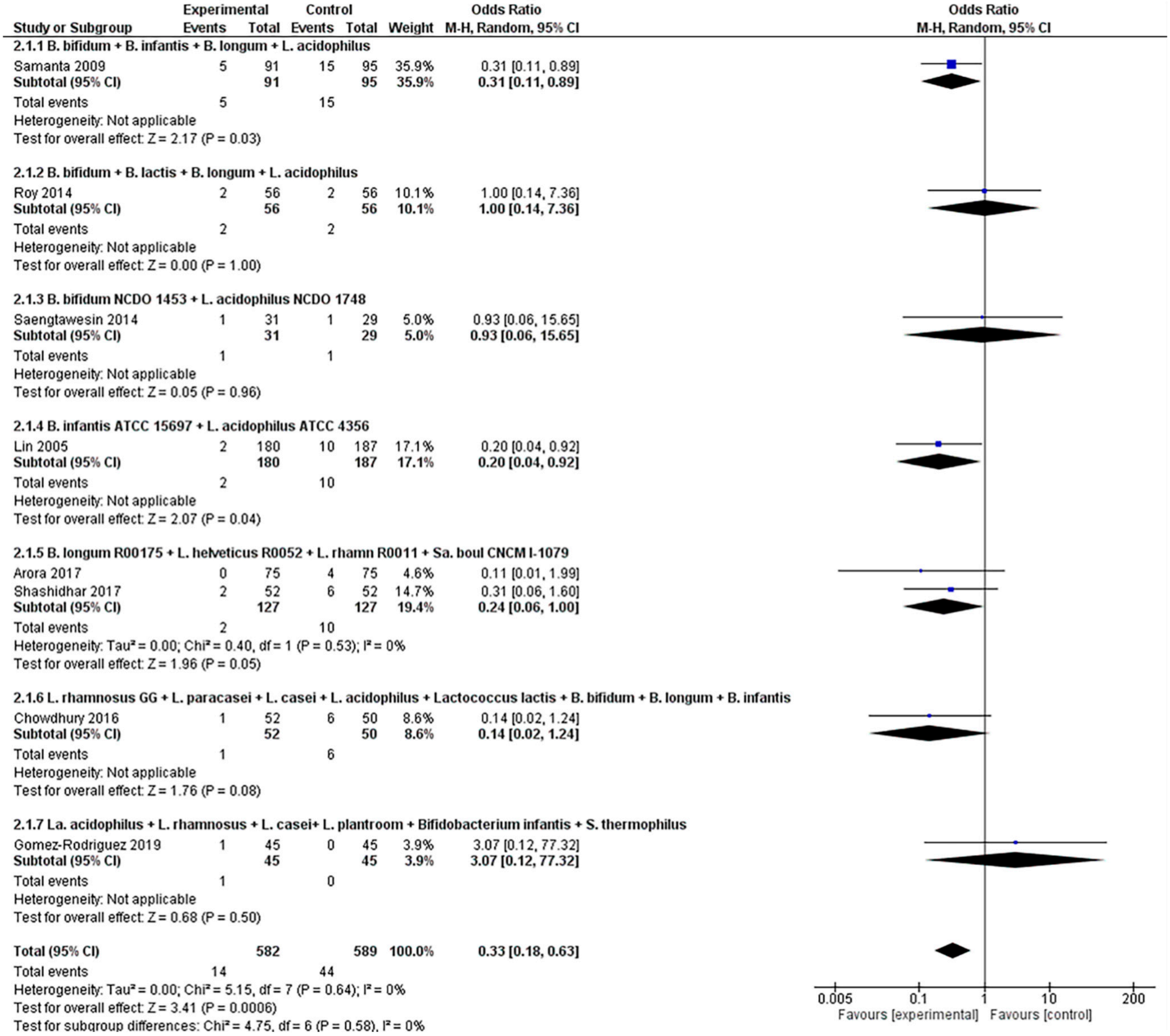

Supplemental Figure 11. Classic pair-wise forest plot showing the association between the use of single probiotics treatment included in the MG group and NEC all stages in 8 studies reporting data for exclusively human milk-fed preterm infants. The study by Gomez- Rodriguez et colleagues is a head-to-head comparison between the multi genus probiotic above and the single strain *L. acidophilus* LB

Supplemental Table 1. Probiotics intervention and corresponding treatment category

Primary network meta-analysis		Subgroup network meta-analysis by infant feeding	
**Treatment category**	**Probiotic**	**Treatment category**	**Probiotic**
B.b	B. breve BBG YIT4010	·	
B.b	B. breve BBG-001	B.bM16V	B. breve M-16V
B.b	B. breve M-16V	·	
B.la	B. lactis Bb-12 OR B. lactis B94	B.la	B. lactis Bb-12 OR B. lactis B94
B.lBB536	B. longum BB536	B.lBB536	B. longum BB536
L.r	L. reuteri DSM 17938	L.r 17938	L. reuteri DSM 17938
L.r	L. reuteri ATCC 55730	·	
LGG 53103	L. rhamnosus GG ATCC 53103	LGG 53103	L. rhamnosus GG ATCC 53103
L.a	L. acidophilus LB	L.ab	L. acidophilus LB
LCR35	L.casei var. rhamnosus (LCR 35)	LCR35	L. casei var. rhamnosus (LCR 35)
Ba.co	Ba. coagulans (L sporogenes)	·	
Ba.c	Ba. clausii (4 strains)	Ba.c	Ba. clausii (4 strains)
Sa.b	Sa. boulardii CNCM I-745	Sa.b	Sa. boulardii CNCM I-745
Sa.b	Sa. boulardii CNCMI-3799	·	
B.MS	B. lactis Bb-12 + B. longum BB536	B.MS	B. lactis Bb-12 + B. longum BB536
B.l+LGG	B. longum 35624 + L. rhamnosus GG	B.l+LGG	B. longum 35624 + L. rhamnosus GG
B.l+LGG	B. longum BB536 + L. rhamnosus GG	·	
MG	B. bifidum + B. infantis + B. longum + L. acidophilus	MG	B. bifidum + B. infantis + B. longum + L. acidophilus
MG	B. bifidum + B. lactis + B. longum + L. acidophilus	MG	B. bifidum + B. lactis + B. longum + L. acidophilus
MG	B. bifidum NCDO 1453 + L. acidophilus NCDO 1748	MG	B. bifidum NCDO 1453 + L. acidophilus NCDO 1748
MG	B. breve + L. casei	·	
MG	B. infantis ATCC 15697 + L. acidophilus ATCC 4356	MG	B. infantis ATCC 15697 + L. acidophilus ATCC 4356
MG	B. infantis Bb-02 + B. lactis Bb-12 +S. thermophilus TH-4	·	
MG	B. infantis PTA-5843 + E. faecium PTA-5844 + L. gasseri PTA-5845	·	
MG	B. lactis Bb-12 + L. rhamnosus GG	·	
MG	B. longum R00175 + L. helveticus R0052 + L. rhamn R0011 + Sa. boulardii CNCM I-1079	MG	B. longum R00175 + L. helveticus R0052 + L. rhamnosus R0011 + Sa. boulardii CNCM I-1079

MG	L. rhamnosus GG + L. paracasei + L. casei + L. acidophilus + Lactococcus lactis + B. bifidum + B. longum + B. infantis	MG	L. rhamnosus GG + L. paracasei + L. casei + L. acidophilus + Lactococcus lactis + B. bifidum + B. longum + B. infantis
MG	La. acidophilus + L. rhamnosus + L. casei + L. plantarum + B. infantis + S. thermophilus	MG	La. acidophilus + L. rhamnosus + L. casei + L. plantarum + B. infantis + S. thermophilus

Supplemental Table 2. List of double-zero trials excluded from primary network meta-analysis

Study	Infant feeding information	Arms	Events	Patients	Comparator	Treatment	Treatment category
Fujii 2006	No	2	0	19	Placebo	B. breve M-16V	B.b
Kitajima 1997	No	2	0	91	Placebo	B. breve BBG YIT4010	B.b
Oshiro 2019	Yes	2	0	34	Placebo	B. breve BBG-001	B.b
Romeo 2011	No	3	0	166	Placebo	L. reuteri ATCC 55730 / L. rhamnosus GG ATCC 53103	L.r/ LGG 53103
Totsu 2014	No	2	0	283	Placebo	B. bifidum OLB6378	B.bi
Umezaki 2010	Yes	2	0	208	Placebo	B. breve M-16V	B.b
Wang 2007	Yes	2	0	66	Placebo	B. breve M-16V	B.b
Xu 2016	Yes	2	0	100	Placebo	Sa. boulardii CNCM I-745	Sa.b
Zeber-Lubecka 2016	No	2	0	39	Placebo	Sa. boulardii CNCMI-3799	Sa.b

Supplemental Table 3. Assessment of the body of evidence according to the GRADE working group approach

Study	Levels of quality of evidence in the GRADE approach
Al-Hosni, 2012	LOW
Arora, 2017	LOW
Awad, 2010	LOW
Bin-Nun, 2005	VERYLOW
Braga, 2011	HIGH
Chowdhury, 2016	MODERATE
Costalos, 2003	HIGH
Costeloe, 2016	HIGH
Cui, 2019	MODERATE
Dani, 2002	MODERATE
Demirel, 2013	MODERATE
Dilli, 2015	MODERATE
Dongol Singh, 2017	MODERATE
Dutta, 2015	LOW
Fernàndez-Carrocera, 2013	HIGH
Fuji, 2006	LOW
Gòmez-Rodriguez, 2019	MODERATE
Hays, 2016	HIGH
Hernandez-Enriquez, 2016	LOW
Jacobs, 2013	LOW
Kaban, 2019	MODERATE
Kanic, 2015	LOW
Kitajima, 1997	MODERATE
Lin, 2005	HIGH
Lin, 2008	HIGH
Manzoni, 2006	MODERATE
Mihatsch, 2010	MODERATE
Mohan, 2006	LOW
Oncel, 2013	MODERATE
Oshiro, 2019	LOW
Patole, 2014	HIGH
Rojas, 2012	HIGH
Romeo, 2011	MODERATE
Rougé, 2009	LOW
Roy, 2014	MODERATE
Saengtawesin, 2014	LOW
Samanta, 2009	MODERATE
Sari, 2011	MODERATE
Serce, 2013	MODERATE
Shadkam, 2015	LOW
Shashidhar, 2017	LOW
Stratiki, 2007	LOW
Tewari, 2016	HIGH
Totsu, 2014	MODERATE
Umezaki, 2010	MODERATE
Usman, 2018	MODERATE
Van Niekerk, 2015	HIGH
Wang, 2007	MODERATE
Wejryd, 2018	HIGH
Xu, 2016	MODERATE
Zeber-Lubecka, 2016	MODERATE

Supplemental Table 4. Results from frequentist network meta-analysis based on electrical network theory. *P*-scores measure the certainty that one treatment is better than another treatment, averaged over all competing treatments, and are equivalent to the posterior means of SUCRA scores from Bayesian network meta-analysis.

**Treatment**	***P*-score**
L.a	0.981
MG	0.753
B.la	0.749
B.lBB536	0.712
L.r	0.643
LCR35	0.554
Ba.co	0.472
LGG 53103	0.452
Sa.b	0.438
B.l+LGG	0.383
Ba.c	0.304
B.b	0.237
P	0.179
B.MS	0.143
